# Perivascular adipose tissue and vascular inflammation: from biological insights to clinical implications

**DOI:** 10.3389/fphys.2026.1766197

**Published:** 2026-01-15

**Authors:** Hiroyuki Sowa, Kohei Karasaki, Mai Ishiwata, Xu Cheng, Masaki Hashimoto, Kazutaka Ueda

**Affiliations:** 1 Department of Cardiovascular Medicine, Graduate School of Medicine, The University of Tokyo, Tokyo, Japan; 2 Cardiovascular Biobank Research Center, International University of Health and Welfare, Tokyo, Japan

**Keywords:** browning/beiging, cardiovascular, FAI (fat attenuation index), PVAT (perivascular adipose tissue), vascular inflammation

## Abstract

Perivascular adipose tissue (PVAT) has emerged as an active paracrine and metabolic organ that modulates vascular function in both humans and rodents, rather than serving merely as structural support. Vascular inflammation is a central mechanism driving cardiovascular diseases such as atherosclerosis, representing a maladaptive response to vascular injury. Recent evidence indicates that PVAT actively participates in this process through dynamic phenotypic changes, including adipose tissue browning or beiging. Furthermore, advances in imaging have enabled the noninvasive evaluation of vascular inflammation using computed tomography–derived indices that reflect PVAT characteristics. This review summarizes current understanding of the interplay between PVAT and vascular inflammation, highlights the biological and clinical implications of PVAT remodeling, and discusses emerging diagnostic approaches and future research directions.

## Introduction

Atherosclerosis has traditionally been viewed as a disease initiated by endothelial injury (Ross and Glomset, 1976; Rader and Daugherty, 2008; Silvestre-Roig et al., 2020), with therapeutic efforts primarily directed toward the vascular intima (Aikawa et al., 2002; Libby et al., 2009; Gimbrone and García-Cardeña, 2016). However, accumulating evidence has revealed that perivascular adipose tissue (PVAT) also plays a crucial regulatory role in vascular biology through “outside-in” signaling (Kim et al., 2020). Under physiological conditions, PVAT contributes to vascular homeostasis by releasing anti-inflammatory and vasodilatory factors. In contrast, during metabolic dysfunction, chronic inflammation, or aging, PVAT becomes a source of proinflammatory mediators that promote atherosclerosis progression in cooperation with immune cells accumulating in the perivascular region (Takaoka et al., 2009). Interestingly, not all individuals with excess adiposity develop metabolic disorders such as type 2 diabetes, dyslipidemia, or hypertension—a phenotype described as metabolically healthy obesity. This condition has been linked to a more favorable inflammatory profile within PVAT (Costa et al., 2018), suggesting that adipose tissue quality may be as critical as its quantity in maintaining vascular health.

Recently, growing interest has focused on quantitative assessment of PVAT inflammation, particularly through computed tomography (CT)–based imaging approaches (Antonopoulos and Antoniades, 2018), which offer noninvasive insights into vascular pathophysiology and may enable refined cardiovascular risk stratification. This review highlights recent advances in PVAT biology and explores its emerging clinical implications in atherosclerotic disease.

## Anatomical characteristics of PVAT

Arteries are traditionally described as having three concentric layers: the tunica intima, tunica media, and tunica adventitia. The adventitia, the outermost layer, consists primarily of collagen and elastic fibers and contains the vasa vasorum, a microvascular network that supplies the arterial wall. Beneath the adventitia lies the media, composed mainly of smooth muscle cells interlaced with collagen and elastic fibers, and separated from the intima by the internal elastic lamina. The intima, the innermost layer, is formed by endothelial cells and bounded externally by the internal elastic lamina (Tellides and Pober, 2015).

**FIGURE 1:**
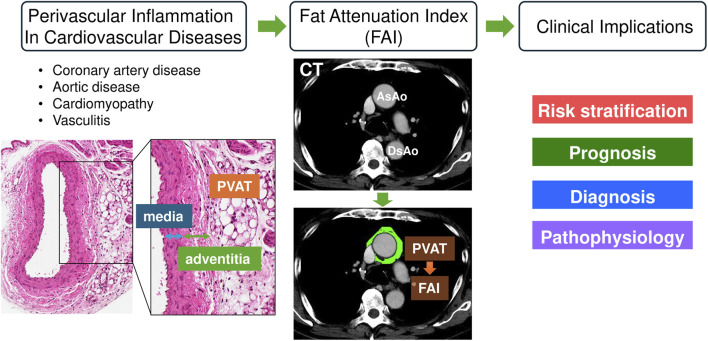
The utility of fat attenuation index (FAI) on cardiovascular disease. Blood vessels consist of three distinct layers: the intima, media, and adventitia. Surrounding the adventitia is the perivascular adipose tissue (PVAT), which lacks a well-defined anatomical boundary separating it from the adventitial layer. Fat attenuation index (FAI) refers to the CT value of PVAT and can be used to assess the state of vascular inflammation. FAI can be applied to risk stratification, prognosis, diagnosis, and elucidation of the pathophysiology of various cardiovascular diseases, and its further utilization is anticipated in the future. The green area on the CT image represents the PVAT region identified by CT values. AsAo, ascending aorta; CT, computed tomography; DsAo, descending aorta.

Adipose tissue is distributed throughout the body in specific depots, and the adipose layer surrounding arteries and veins with a diameter of approximately 100 μm or greater is referred to as PVAT(Henrichot et al., 2005). Recent studies define PVAT as a layer of adipocytes extending a distance from the vascular wall equivalent to the vessel’s internal diameter. For larger vessels, such as the aorta, PVAT is defined as the adipose layer extending up to 2 cm from the external surface of the vessel wall (Mazurek et al., 2017; Antonopoulos and Antoniades, 2018; Goeller et al., 2018; Oikonomou et al., 2019; Oikonomou et al., 2020; Dai et al., 2020; Bengs et al., 2021). Most systemic arteries are enveloped by PVAT, except for cerebral and pulmonary vessels, where it is absent (Xiong et al., 2018). Importantly, there is no clear anatomical boundary between the adventitia and PVAT; rather, PVAT is continuous with the adventitial layer, forming a structural and functional continuum between the vessel wall and surrounding adipose tissue (Figure 1).

## Molecular characteristics of PVAT

In mammals, adipose tissue is broadly classified into white adipose tissue (WAT) and brown adipose tissue (BAT) (Virtanen et al., 2009; Frontini and Cinti, 2010; Harms and Seale, 2013; Rosen and Spiegelman, 2014). WAT is composed of large unilocular adipocytes with sparse cytoplasm and primarily functions as an energy reservoir by storing triglycerides, which can be mobilized through lipolysis to release free fatty acids. In contrast, BAT contains multilocular adipocytes enriched with mitochondria expressing uncoupling protein 1 (UCP1) (Kajimura et al., 2015; Wang and Seale, 2016). UCP1, located in the inner mitochondrial membrane, mediates thermogenesis by uncoupling oxidative phosphorylation from adenosine triphosphate (ATP) synthesis (Yau and Yen, 2020). Notably, WAT can acquire BAT-like features in response to specific stimuli such as cold exposure or hormonal activation, giving rise to “beige” adipocytes through a process known as beiging (Wu et al., 2012; Sidossis and Kajimura, 2015).

Adipocytes within PVAT exhibit heterogeneous phenotypes depending on vascular location. In mice, thoracic aortic PVAT displays BAT-like morphological and functional features (Fitzgibbons et al., 2011), whereas PVAT surrounding the carotid, femoral, and mesenteric arteries resembles WAT. Abdominal aortic PVAT shows intermediate characteristics, suggesting a beige-like phenotype (Brown et al., 2014; Horimatsu et al., 2017). In humans, PVAT also shows regional heterogeneity. For example, gene expression profiling demonstrates distinct PVAT transcriptomes surrounding coronary versus internal thoracic arteries, indicating depot-specific differences in inflammation and metabolism in human PVAT (Lu et al., 2017). Moreover, PVAT from the internal thoracic artery exhibits higher expression of thermogenic genes such as UCP1 compared with subcutaneous fat, supporting the existence of brown/beige-like features in certain human vascular beds (Kowalówka et al., 2020). Imaging and post-mortem studies further suggest that thoracic and periaortic PVAT may have more thermogenic characteristics, whereas abdominal and peripheral depots are more WAT-like (Queiroz and Sena, 2024). Although direct extrapolation from rodents to humans requires caution, these observations suggest that key principles of PVAT heterogeneity and plasticity are conserved across species. Moreover, PVAT is also highly plastic; for example, prolonged exposure to moderate cold (16 °C) induces a BAT-like phenotype in thoracic PVAT, with upregulation of UCP1 and PGC1α/β, changes thought to exert protective effects against atherosclerosis (Chang et al., 2012).

Lineage-tracing studies have revealed distinct developmental origins for different adipocyte subtypes. White adipocytes derive from mural progenitors expressing *Cd24*, *Cd34*, and *Pdgfra* (Rodeheffer et al., 2008), while classical brown adipocytes arise from dermomyotomal precursors expressing *Pax7*, *Engrailed-1*, and *Myf5* (Timmons et al., 2007). Because MyoD is a key myogenic transcription factor expressed during skeletal muscle differentiation, its involvement has been used to delineate the timing of lineage commitment toward brown adipogenesis. As MyoD-expressing cells do not differentiate into brown adipocytes, lineage commitment toward the brown adipocyte fate occurs prior to MyoD activation (Seale et al., 2008; An et al., 2017). Both WAT and BAT originate from mesodermal mesenchymal stem cells; however, WAT is *Myf5*-negative and likely arises from sclerotome-derived precursors (Wang and Seale, 2016). Beige adipocytes appear to have heterogeneous developmental origins depending on their anatomical depot, arising from precursor populations expressing *Tagln*, *Myh11*, *Pdgfra*, or *Pdgfrb* (Long et al., 2014). In some depots, beige adipocytes may also derive from *Pax3*- or *Myf5*-expressing progenitors (Sanchez-Gurmaches and Guertin, 2014), suggesting multiple developmental pathways for beige fat formation.

Recent studies indicate that PVAT possesses distinct molecular and developmental characteristics compared with both WAT and BAT, and that its developmental origin varies across vascular beds. Single-cell transcriptomic analyses show that murine thoracic PVAT originates from fibroblast-like precursors (*Pdgfra* ^+^, *Ly6a* ^+^, *Pparg* ^+^) transcriptionally similar to WAT-derived cells (Li et al., 2021). In contrast, adipogenic smooth muscle–like cells within the aortic adventitia (*Myh11* ^+^, *Pdgfra* ^−^, *Pparg* ^+^) may also contribute to PVAT adipogenesis. Human PVAT similarly contains both fibroblast-like and smooth muscle–like progenitors (Angueira et al., 2021). Proteomic profiling demonstrates that thoracic aortic PVAT shares only partial similarity with either BAT (43% overlap) or visceral WAT (44%), while the overlap between BAT and WAT is 53%. Collectively, these findings underscore that PVAT represents a distinct adipose depot with unique molecular, cellular, and developmental features (Boucher et al., 2020).

## The pathophysiological roles of PVAT

PVAT surrounds most blood vessels and was traditionally regarded as providing structural support and thermal insulation. However, experimental studies in rats demonstrated that removal of PVAT attenuates vascular contractility, indicating that PVAT actively contributes to vascular regulation (Soltis and Cassis, 1991). PVAT secretes a wide array of bioactive substances—including adipokines, both anti- and pro-inflammatory cytokines, microRNAs, hydrogen sulfide, reactive oxygen species, and fatty acid metabolites—that play pivotal roles in maintaining vascular homeostasis (Szasz et al., 2013).

Under physiological conditions, PVAT exerts vasodilatory, antioxidant, and anti-inflammatory effects on the vasculature. These actions are mediated through both direct modulation of vascular smooth muscle tone and enhancement of endothelial-derived mediators such as nitric oxide (NO) and endothelium-derived hyperpolarizing factor (Gao et al., 2007; Victorio et al., 2016; Almabrouk et al., 2017; Agabiti-Rosei et al., 2018). Activation of AMP-activated protein kinase (AMPK) and stimulation of NO signaling are thought to be key pathways underlying these protective effects (Salt and Hardie, 2017; Almabrouk et al., 2018; Hwej et al., 2024). Among PVAT-derived adipokines, adiponectin is the most extensively studied. Since its discovery in 1995, numerous studies have shown that adiponectin promotes pancreatic β-cell survival, enhances insulin sensitivity, and exerts renoprotective and cardioprotective effects (Okamoto et al., 2000; Lam and Xu, 2005; Tilg and Moschen, 2006; Ohashi et al., 2011). In the vasculature, adiponectin maintains endothelial integrity through AMPK-dependent activation of endothelial NO synthase (eNOS), vasorelaxant activity, and anti-inflammatory actions (Okamoto et al., 2000; Fésüs et al., 2007; Zhu et al., 2008).

Under metabolic stress conditions—such as obesity, hypertension, insulin resistance, or aging—PVAT undergoes phenotypic changes that promote inflammation (Xia et al., 2016; Almabrouk et al., 2018; da Costa et al., 2018; Nakladal et al., 2022). The inflamed PVAT shifts toward the secretion of pro-inflammatory and pro-atherogenic mediators, including interleukin (IL)-1β, IL-6, IL-8, tumor necrosis factor-α (TNF-α), and monocyte chemoattractant protein-1 (MCP-1), which contribute to endothelial dysfunction, vascular insulin resistance, and increased vascular tone (Koenen et al., 2021). PVAT under pathological conditions has also been reported to harbor inflammatory cells, including macrophages, T cells, and B cells, which could be associated with plaque instability, vascular aging, and hypertension (Farias-Itao et al., 2019; Berillo et al., 2024; Tai et al., 2025). Moreover, the residential cells in PVAT are also possibly changed in response to disease conditions. In PVAT around abdominal aortic aneurysm, perivascular stromal cells, which normally differentiate to perivascular adipocytes, reportedly differentiate to myofibroblasts, which may induce fibrotic remodeling of PVAT and aneurysm developments (Pan et al., 2025). Overall, PVAT exposed to pathological stress changes in multiple aspects, including transcriptomic profiles, cellular composition and tissue structure, possibly leading to the acquisition of a disease-promoting phenotype.

Collectively, PVAT exhibits a paradoxical nature: while it maintains vascular homeostasis under physiological conditions through anti-inflammatory and protective mechanisms, metabolic stress induces its transformation into a pathogenic phenotype that exacerbates vascular injury. This phenotypic shift may be partly driven by alterations in its thermogenic and beige-like characteristics.

## Beiging of PVAT in response to vascular injury

PVAT is composed of heterogeneous cell populations, with mature adipocytes accounting for roughly half of its volume, and the remainder consisting of immune cells, fibroblasts, adipocyte progenitors, vascular cells, mesenchymal stem-like cells, and nerve fibers (Silva et al., 2017; Kumar et al., 2020). Crosstalk among these cellular components maintains vascular homeostasis but may also contribute to disease progression. Notably, PVAT surrounding human coronary arteries undergoes phenotypic remodeling in response to vascular inflammation (Antonopoulos et al., 2017), suggesting a bidirectional interaction between PVAT and the vessel wall, although the underlying mechanisms remain incompletely defined.

To elucidate the relationship between PVAT remodeling and vascular injury, we employed a mouse model of wire-induced femoral artery injury (Adachi et al., 2022). Transcriptomic analysis of perivascular tissue revealed marked upregulation not only of inflammatory genes but also of brown adipose tissue (BAT)-related genes such as *Ucp1*, *Cidea*, and *Cox8b*. These findings, confirmed by multiple complementary techniques, demonstrated that vascular injury triggers a brown fat–like activation, or “beiging,” within PVAT. This response was abolished in adipose-specific *Prdm16* knockout mice, which exhibited exacerbated inflammation and vascular remodeling. Similarly, local *Prdm16* knockdown by siRNA enhanced PVAT inflammation, whereas pharmacological stimulation of β3-adrenergic signaling using CL316243 promoted PVAT beiging and ameliorated vascular remodeling.

Conditioned medium experiments further suggested that beige PVAT secretes anti-inflammatory mediators capable of modulating macrophage polarization. Single-cell RNA sequencing identified *Neuregulin 4* (*Nrg4*) as one such adipokine, and *Nrg4* knockdown abrogated the anti-inflammatory effects of beige PVAT, implicating the NRG4 signaling pathway as a key in this process.

In human aortic samples, PVAT from patients with aortic dissection displayed smaller lipid droplets and increased UCP1 expression compared with control PVAT, consistent with a beiging phenotype. Moreover, expression of NRG4 was markedly higher in PVAT adjacent to dissected aortas. Collectively, these findings indicate that PVAT beiging occurs as an adaptive response to acute vascular injury in both mice and humans, contributing to the transition from the initial inflammatory phase toward resolution and repair.

## PVAT as the potential therapeutic target for cardiovascular disease

Therapeutic strategies targeting PVAT function are gaining attention. Several antidiabetic agents exert cardiovascular protective effects beyond glycemic control (Marso et al., 2016b; Marso et al., 2016a; Gerstein et al., 2019), partly by modulating PVAT and epicardial adipose tissue (EAT). Because EAT is mainly distributed around the coronary arteries, it can be regarded as largely corresponding to coronary PVAT (Morano et al., 2015; Dutour et al., 2016; Iacobellis and Villasante Fricke, 2020). GLP-1 receptor (GLP1R) agonists reduce cardiovascular events and decrease EAT thickness, likely via direct effects on GLP1R-expressing adipose tissue. These agents also promote adipose beiging and suppress adipogenesis (Dozio et al., 2019; Iacobellis, 2022). The dual GIP/GLP1R agonist tirzepatide further improves outcomes in obese patients with heart failure with preserved ejection fraction (HFpEF), accompanied by reductions in paracardial fat (Kramer et al., 2025; Packer et al., 2025). Similarly, SGLT2 inhibitors lower cardiovascular risk in both diabetic and non-diabetic populations (Zinman et al., 2015; Wiviott et al., 2019), reduce EAT volume (Fukuda et al., 2017; Yagi et al., 2017; Sato et al., 2018; Iacobellis and Gra-Menendez, 2020; Requena-Ibáñez et al., 2021), and enhance lipolysis (Iacobellis, 2022). These antidiabetic agents have been shown to attenuate PVAT inflammation related to diabetes in preclinical studies (Mori et al., 2019; Chen et al., 2021). Furthermore, the β3-adrenergic receptor signaling has been indicated to shift the PVAT phenotype toward a vascular protective state. The pharmacological stimulation of the β3-adrenergic receptor in mouse models of wire-induced vascular injury induces PVAT beiging which attenuates arterial inflammation (Adachi et al., 2022). In a mouse model of aortic dissection, mirabegron, a β3-adrenergic receptor agonist, reduces PVAT inflammation and disease severity likely through promoting lymphangiogenesis in PVAT, which can enhance the drainage of inflammatory cells (Zhang et al., 2024). These evidence consistently suggest PVAT as a promising therapeutic target for vascular diseases. Further investigation into PVAT-mediated therapeutic mechanisms may yield insights into the development of novel treatment strategies.

## Fat attenuation index (FAI): a novel imaging biomarker for PVAT inflammation

^18^F-fluorodeoxyglucose positron emission tomography (^18^F-FDG PET) has long been recognized as a valuable tool for assessing inflammatory activity within adipose tissue (Christen et al., 2010; Bucerius et al., 2014). However, its high cost and limited availability have constrained its routine clinical use, despite its proven utility in research and selected clinical applications. These limitations highlight the need for alternative, more accessible imaging modalities to evaluate adipose tissue inflammation non-invasively.

In this context, computed tomography angiography (CTA) has recently emerged as a promising technique to quantify PVAT inflammation associated with vascular injury (Antonopoulos et al., 2017). The FAI, derived from CT imaging, was developed to characterize the radiological signature of inflamed adipose tissue by analyzing human coronary PVAT. Clinical studies have demonstrated that elevated coronary FAI values reflect vascular inflammation, particularly in acute coronary syndromes (ACS), and can identify vulnerable atherosclerotic plaques with meaningful sensitivity and specificity (Antonopoulos et al., 2017). Evidence from the Cardiovascular RISk Prediction using Computed Tomography (CRISP-CT) study established the prognostic value of FAI, showing that increased coronary FAI independently predicts all-cause and cardiac mortality in large patient cohorts (Oikonomou et al., 2018). Moreover, subsequent investigations confirmed that FAI enhances cardiovascular risk stratification beyond conventional risk factors and standard CTA parameters (Oikonomou et al., 2018).

In ACS, elevated FAI corresponds to localized inflammatory burden at culprit lesions. Notably, vascular inflammation resolves to baseline in approximately 80% of patients within 6 months, whereas persistent inflammation in the remaining 20% is linked to a markedly higher risk of recurrent events (Antonopoulos et al., 2017; Oikonomou et al., 2019; Antoniades et al., 2020). FAI around coronary arteries with stent placement has been reported to be an independent predictor of in-stent restenosis after percutaneous coronary intervention, suggesting that FAI may be useful for evaluating the risk of inflammation-related vascular events (Qin et al., 2022) (Table 1).

**TABLE 1:** Research on cardiovascular disease utilizing FAI.

Category	Disease	Key findings	References
Risk stratification	ACS	Incorporating FAI into ACS risk stratification improves MACE prediction beyond conventional methods	Antonopoulos et al. (2017) Oikonomou et al. (2019) Antoniades et al. (2020)
	ACS	FAI around coronary arteries with stent placement is an independent predictor of in-stent restenosis after PCI.	Qin et al. (2022)
	AAD	A higher FAI is a risk factor for all-cause death and aortic events in uncomplicated TBAD patients	Adachi et al. (2025)
Prognosis	AAA	Elevated aortic FAI is associated with abdominal aortic aneurysm expansion	Yamaguchi et al. (2021)
	Carotid artery plaque	Increased pericarotid FAI serves as an indirect marker of carotid plaque instability	Hu et al. (2021) Yu et al. (2022)
	MFS	FAI correlates with the expansion speed of the ascending aortic diameter in MFS patients	Sowa et al. (2025)
Diagnosis	AAD	FAI and D-dimer show comparable diagnostic capability for AAD.	Adachi et al. (2025)
Pathophysiology	MFS	Perivascular inflammation contributes to aneurysm formation in MFS patients	Sowa et al. (2025)

AAA, abdominal aortic aneurysm; AAD, acute aortic dissection; ACS, acute coronary syndrome; FAI, fat attenuation index; MACE, major adverse cardiovascular events; MFS, marfan syndrome; PCI, percutaneous coronary intervention; TBAD, type B aortic dissection.

## Potential of FAI across multiple vascular beds

Emerging research has extended FAI assessment beyond the coronary arteries to other vascular territories. In the carotid artery, pericarotid fat density on the stroke-affected side was significantly higher than on the contralateral side in patients with embolic stroke of undetermined source, suggesting perivascular inflammation extending beyond the arterial wall (Hu et al., 2021). Furthermore, increased pericarotid fat density has been proposed as an indirect marker of carotid plaque instability (Yu et al., 2022). Similarly, studies of the abdominal aorta have reported that elevated aortic FAI values are associated with aneurysm expansion (Yamaguchi et al., 2021) (Table 1).

The clinical significance of periaortic FAI has also been investigated in acute aortic dissection (AAD). In a multicenter retrospective study involving 135 patients with uncomplicated type B AAD, higher periaortic FAI values on noncontrast CT—peaking around day 6 after onset—were independently associated with an increased risk of death and aortic events (hazard ratio, 4.54; P = 0.003) (Adachi et al., 2025). These data support FAI as a promising prognostic biomarker reflecting vascular inflammatory activity.

Our research has also focused on the contribution of inflammatory adipose tissue to vascular pathology in Marfan syndrome (MFS), a hereditary aortic disorder (Sowa et al., 2025). Both human and murine studies have revealed enhanced inflammatory activity within PVAT of MFS. Metabolic inflammatory stress induced by a high-fat diet (HFD) further amplified vascular inflammation, predominantly in periaortic regions, and accelerated aortic dilation in *Fbn1* ^C1041G/+^ mice. These effects were significantly attenuated by low-dose pitavastatin. HFD feeding also aggravated medial disorganization in the aortic wall, accompanied by increased activation of TGF-β downstream targets, which were likewise mitigated by pitavastatin treatment. We quantified the perivascular FAI of the ascending aorta (AA-FAI) as an imaging correlate of periaortic inflammation. AA-FAI values were significantly elevated in patients with MFS compared with those without hereditary connective tissue disorders (Sowa et al., 2025). Collectively, these findings suggest that perivascular inflammation contributes to aneurysm formation in MFS and represents a potential therapeutic target for preventing vascular complications.

Several clinical studies have demonstrated reductions in FAI following statin therapy and after administration of anti-inflammatory agents for conditions such as psoriasis and IgG4-related disease (Elnabawi et al., 2019; Du et al., 2023). Furthermore, the CANTOS trial established that targeting inflammation with an anti–IL-1β monoclonal antibody reduces recurrent cardiovascular events in post–myocardial infarction patients with sustained inflammation, underscoring the therapeutic potential of inflammation suppression (Ridker et al., 2017).

Altogether, accumulating evidence indicates that FAI not only provides a noninvasive measure of perivascular inflammation but may also enhance cardiovascular risk stratification and guide the cost-effective implementation of anti-inflammatory therapies in vascular disease management (Figure 1).

## Discussion

PVAT is more than a structural support; it functions as an active endocrine organ that critically modulates vascular inflammation via “outside-in” signaling. Under physiological conditions, PVAT promotes vascular homeostasis through the secretion of anti-inflammatory and vasodilatory factors. Conversely, metabolic stress induces PVAT inflammation, leading to the release of pro-inflammatory cytokines such as IL-1β, IL-6, and TNF-α, which contribute to endothelial dysfunction and vascular insulin resistance. Notably, PVAT beiging following vascular injury appears to facilitate the resolution of local inflammation. As exemplified by evidence that the cardioprotective effects of GLP-1 receptor agonists and SGLT2 inhibitors have been shown to be mediated, in part, through their actions on PVAT and EAT, research focusing on PVAT function is expected to contribute to advances in cardiovascular pathophysiology and the development of novel therapeutic approaches.

CT–derived measurements, particularly the FAI, provide a noninvasive, quantitative assessment of PVAT inflammation. Initially validated in coronary PVAT, FAI has shown potential to extend to extracoronary vascular beds, offering novel insights into vascular pathophysiology and disease monitoring. Elevated coronary FAI has been reported in conditions such as takotsubo cardiomyopathy, correlating with worse prognosis independent of conventional risk factors (Sayama et al., 2023), further highlighting the role of vascular inflammation in disease progression. Overall, FAI represents a promising, reproducible imaging biomarker for assessing perivascular inflammation. Further studies are needed to standardize measurement protocols and validate its application across diverse populations and vascular territories, and continued research in this area may advance both mechanistic understanding and therapeutic strategies for inflammatory vascular diseases.

In conclusion, PVAT is an active regulator of vascular homeostasis, capable of both protective and deleterious effects depending on metabolic and inflammatory states. Recent emerging evidence underscore PVAT as both a key contributor to vascular disease and a promising target for intervention. Future research should focus on elucidating the molecular mechanisms governing PVAT plasticity, standardizing FAI assessment across vascular beds, and translating these insights into therapeutic strategies to mitigate cardiovascular risk.
